# Improving environmental cleaning process in hemodialysis unit at King Saud Medical City

**DOI:** 10.1186/2047-2994-4-S1-P67

**Published:** 2015-06-16

**Authors:** HA Amer, H Amer, D Kumar, H Alzoman, N Gul, A Hossam, S Ali

**Affiliations:** 1 Infection Control Dept , King Saud Medical City , Riyadh, Saudi Arabia

## Introduction

Maintenance of a clean environment at health care facilities is an important factor in reducing the risk of cross Infection. Unmonitored organic residues can build up to high levels on surfaces if adequate cleaning procedures are not adopted. Currently there is no consistent methodology to evaluate environmental cleaning process at king Saud Medical City. Our project aimed to develop monitoring tools to ensure proper surfaces cleaning. Project was implemented at Hemodialysis unit as it provides care for immunocompromised patients at high risk acquiring infections.

## Objectives

Develop monitoring tools.

Increase % of high touch surfaces cleaning by 90% from base line.

Improve cleaning quality.

## Methods

FOCUS-PDCA PIP was conducted to improve the cleaning process in between dialysis sessions strategies include:

1. Educate Nurses & Housekeepers and distribute Flowchart standardize the Cleaning Process.

2. Monitoring tools:

a. Adenosine Triphosphate (ATP) assay that measures organic residues on the surfaces presented by Quantified Relative Light Units (RLU)]. The lower the RLU value The higher the cleaning level. As per instrument user manual, test reading of 500 RLUs consider accepted level for cleaning. Four High Touch Surfaces selected for swabbing after cleaning (Remote Control unit, Bed Rail, Bedside Table, Machine screen) as they are frequently missed.

b. Direct observation Checklist observing cleaning of all patient cubic surfaces using hospital approved disinfectant and proper contact time.

3. Train observers.

4. Control patient entry to dialysis session allowing enough time for cleaning.

5. Reward staff in area with good monitoring results.

## Results

Evidences of improved cleanliness of high-touch surfaces after the intervention program:

Reduction of Relative Light Units on high touch surfaces ranges between 80% and 97% along five regular weekly audit with 16 swabs per each audit (table 1 & Graph 1).

Nearly 50% of collected swabs (total is 84 swabs) presented RLU < 500 and classified as “PASS” (table 2).

92.3% improvement in the frequency of cleaning high touch surfaces (table 3).

## Conclusion

1 - ATP readings provided quantitative evidence to monitor cleanliness of high-touch surfaces after the implementation of an intervention program.

2 - Continue monitoring cleanliness trend level is important to maintain the improved standards.

3 - Extend project methodology to other hospital locations.

## Disclosure of interest

None declared.

